# Transport Properties of Gramicidin A Ion Channel in a Free-Standing Lipid Bilayer Filled With Oil Inclusions

**DOI:** 10.3389/fcell.2020.531229

**Published:** 2020-09-04

**Authors:** Harvey Tawfik, Sevde Puza, Ralf Seemann, Jean-Baptiste Fleury

**Affiliations:** Experimental Physics and Center for Biophysics, Universität des Saarlandes, Saarbrücken, Germany

**Keywords:** lipid bilayer, gramicidin, oil, ion channel, conduction

## Abstract

Ion channels are key proteins in mammalian cell membranes. They have a central role in the physiology of excitable cells such as neurons, muscle, and heart cells. They also play a crucial role in kidney physiology. The gramicidin ion channel is one of the most studied ion channels, in particular it was intensively employed to investigate the lipid–protein interactions in model cell membranes. For example, even though the sequence of gramicidin is extremely hydrophobic, its motion is impaired in membrane bilayer, i.e., it does not rapidly flip to the other membrane leaflet, and low channel activity were observed when gramicidin is added asymmetrically to only one leaflet of a model cell membrane. In this article, we study the transport properties of gramicidin channel in a heterogeneous model membrane. Using microfluidics, we are forming freestanding bilayers as model cell membranes including heterogeneous domains that are created by oil inclusions. The presence of oil inclusions is then demonstrated by measuring the bilayer capacity via a patch-clamp amplifier and fluorescent confocal inspection. Based on electrophysiological and optical measurements Gramicidin A (gA) ion channels are dispersed into the buffer phases on both side of the formed lipid bilayer and insert spontaneously into the bilayer upon formation. The presence of functional Gramicidin A is then demonstrated by measuring conductivity signals. Based on electrophysiological and optical measurements, we explore the consequence of the presence of these oil inclusions on the functionality of incorporated gA ion channels. For low oil concentration, we measure a decrease of gA transport properties due to the reduction of the bilayer tension. For large oil concentration, we measure a saturation of gA transport properties due to an increase of the bilayer thickness.

## Introduction

Biomembranes are composed of a wide range of biomolecules: lipids, proteins, and cholesterol that are important in several membrane-mediated processes. Functional ion channels formed by transmembrane proteins have a key role in the transport processes of living cells. As example, they have a crucial role in the physiology of excitable cells like neurons, muscle, cardiac cells and in the physiology of kidneys (Kuo and Ehrlich, 2012).

Ion channels enable the transport of small molecules, like ions through bilayer, while protein-free lipid bilayer is impermeable to ionic charges (Miyamoto and Thompson, 1966). Eukaryotic ion channels are pore-forming proteins that allow the flow of ions across membranes, either plasma membranes or the membranes of intracellular organelles (Armstrong, 1975; Hille, 2001; Alexander et al., 2011; Inanobe and Kurachi, 2014). Many ion channels such as most Na, K, Ca and some Cl channels are gated by voltage but others such as certain K and Cl channels, TRP channels, ryanodine receptors and IP_3_ receptors are relatively voltage-insensitive and are gated by second messengers and other intracellular or extracellular mediators. Bacterial ion channels are infecting the host cell to create a bacterial pore, which is eventually damaging host tissues and leading to cells lysis (Armstrong, 1975; Martinac et al., 2008).

Transport properties of bacterial pore ion channels embedded in artificial lipid bilayer have been intensively studied in the past years (Koprowski and Kubalski, 2001; Funakoshi et al., 2006; Bayley et al., 2008; Peraro and van der Goot, 2016; Heo et al., 2019). The obtained results reflect that some particular bacterial channels, like α-hemolysin (Funakoshi et al., 2006; Bayley et al., 2008; Heo et al., 2019), present many advantages compared to mammalian ion channels for model membranes. One of the most studied examples of this type of ion channel is α-hemolysin, a bacterial protein from E-Coli, which is water soluble and which inserts spontaneously into a lipid bilayer that is containing PC phospholipids (Lakey et al., 1994; Diabate et al., 2015; Peraro and van der Goot, 2016). In contrast to mammalian channel proteins, α-hemolysin proteins are easy to synthesize in large quantities and they are water soluble in their monomeric state. Moreover, in their monomeric state, they are spontaneously inserting and diffusing into an artificial lipid bilayer and eventually forming a bacterial ion channel pore by self-assembly (Funakoshi et al., 2006; Bayley et al., 2008; Peraro and van der Goot, 2016; Heo et al., 2019). α-hemolysin pores are composed by 6, or more monomeric proteins (Song et al., 1996). The final pore diameter is depending of the number of individual protein units composing the bacterial-complex. Inorganic monovalent ions, such as potassium or sodium can travel through these pores freely via diffusion (Watanabe et al., 2014). Another example of a highly studied ion channel is Gramicidin A (gA) that consist of a dimeric structure, made of two helices (parallel and anti-parallel) that self-assemble to form a pore protein-complex (Cross et al., 1999). gA can be dispersed in buffer, and like hemolysin, can spontaneously insert into lipid bilayer before diffusing and forming a pore in the bilayer by self-assembly. Here, the self-assembly differs from hemolysin, as each gA monomer occupies only one leaflet of the bilayer, thus it needs two monomers face-to-face to form an ion channel (Cross et al., 1999; Acharya et al., 2013). Such a gA ion channel also enables inorganic monovalent ions, as potassium or sodium to freely travel through these pores via diffusion (Cross et al., 1999). In low doses gA can be used as antibiotic medications against gram-positive bacteria (Urry, 1971; Van Epps, 2006; Wang et al., 2012; Lum et al., 2017). As gA forms only pores that consist of dimers, these pores have a constant diameter, in contrast to hemolysin that can form pores with different diameter and therefore present different transport properties (Ketchem et al., 1993; Lum et al., 2017).

As mentioned previously, lipid bilayer has been employed, intensively, to characterize the conducting properties of gA pores via electrical measurements (Montal and Mueller, 1972). Such planar lipid bilayer is consisting of two separated lipid monolayers stabilized by a solvent is referred to as a Black Lipid Membrane (BLM) (Oiki, 2012). Advantages of the BLM methods over lipid vesicles are the possibility of electrophysiological measurements and the exchange of chemical reagents without the need of tedious vesicle handling and manipulation (Montal and Mueller, 1972; Oiki, 2012; Khangholi et al., 2020). One of the recent approaches with this technique, the droplet interface bilayer (DIB), has been specifically developed to produce a solvent-free lipid bilayer having a rich lipid composition (Bayley et al., 2008). This method also enables a rapid membrane characterization, drug screening, and ion channel recordings, whereas the possibility of having a continuous flow surrounding the bilayer in the DIB technique is somehow limited (Bayley et al., 2008). Thus, many miniaturized apparatuses have been developed, such as microfluidic devices, to overcome the drawbacks of all the previous techniques (Funakoshi et al., 2006). Moreover, the formation of lipid bilayers in microfluidic devices presents the advantage of changing the buffer around the bilayer while enabling good optical and electrophysiological access without using an undesired solvent (typically oil), which is essential for various biotechnological purposes (Heo et al., 2019; Khangholi et al., 2020). However, the properties of these pores in heterogeneous membrane are not known (Cross et al., 1999; Kourie et al., 2002; Acharya et al., 2013).

In this article, we study the transport properties of gA ion channels in heterogeneous membranes. Our heterogeneous membranes consist of a lipid bilayer filled with nanoscopic oil inclusions. It results that this type of heterogeneous bilayer possesses a substantial dynamic heterogeneity due to the diffusion of single oil molecules, or due to the self-organization of nanoscopic oil inclusions (Yang et al., 2016). As oil inclusion, we consider the case of silicone oil (SiAR20 Sigma-Aldrich) which is known to be present in lipid bilayer without forming large scale inclusions (micrometer and larger) (Claudeta et al., 2016). The presence of nanoscopic oil inclusions in our system was confirmed by highly sensitive electrophysiological measurements and confocal fluorescence microscopy. It is in particular of great interest for physicists studying ion channel protein in DiB system (Bayley et al., 2008), because in such systems a massive amount of silicone oil (until 50%) is needed to stabilize the formed lipid bilayer. Thus, it is relevant to investigate if the usage of large silicone oil concentration could affect the transport properties of an ion channel protein.

Behind these physical aspects, studying the influence of silicone oil inclusion in model membranes is also of biological relevance. Indeed, as silicone oils may contaminate the ocean as pollutant, it can be absorbed by algae, plankton, fish or plants (Nendza, 2007). Silicone oil is, also, commonly used in cosmetics and fast food as an anti-foaming agent (Bergeron et al., 1997; Erickson, 2015). Even if silicone oil is typically considered as biologically inert, we investigate its influence on the transport properties of gA ion channels (Bergeron et al., 1997; Erickson, 2015). Moreover, we think that the case of oil spills may not be relevant directly for marine organism, as it is an antibiotic. But it may raises questions for other ion channel proteins with a dimeric structure present in their membrane in presence of an oil pollutant.

Using microfluidics, we are forming a model cell membrane including possible nanodomains consisting of silicone oil inclusions. We explore the consequence of the presence of these oil inclusions on the functionality of incorporated gA ion channel. Our model cell membrane is a free-standing lipid bilayer formed by contacting two aqueous fingers in a microfluidic chip surrounded by an oil phase that contains lipids. Upon pushing the aqueous fingers into the microfluidic device their interfaces get decorated with a lipid monolayer and eventually zip to form a bilayer when the monolayers contact each other (Vargas et al., 2014). The gA ion channels are dispersed into the aqueous buffer phases, on both side of the formed lipid bilayer and insert spontaneously into the bilayer upon bilayer formation. The presence of functional gA channels is demonstrated by measuring conductivity signals via a patch-clamp amplifier. We repeat this study by varying the concentration of silicone oil inside the bilayer. Thus, we explore the effect of silicone oil nano-domains on the conductive properties of gA pores.

## Experimental

### Material and Methods

1,2-Dioleoyl-sn-glycero-3-phosphocholine (DOPC) were bought from Avanti Polar Lipids. DOPC is used because it is one of the most common phospholipids present in human cells (Heo et al., 2019). The silicone oil (SiAR20) and squalene oil were purchased from Sigma-Aldrich. Ultra-pure water was obtained by filtration using a filtration system from Thermo Fisher. PDMS184 was purchased from Dow Corning. Gramicidin A and all other chemical, like salts: LiCl, NaCl, KCl, RbCl, CsCl, were purchased from Sigma-Aldrich. 0.1 M solute solution were prepared by dispersing the salt directly in pure water and under mechanical steering with 1 nM of gA.

### Microfluidic Chips Design and Fabrication

A detailed description of the microfluidic design and fabrication is provided in the Supplementary Material. Two types of chips were produced: a 2D and a 3D microfluidic chips were produced. The 2D microfluidic chip allows the production of a lipid bilayer with a vertical orientation. The 3D microfluidic chip allows the production of a lipid bilayer with a horizontal orientation.

### Surface Tension Measurements and Membrane Tension Calculation

**FIGURE 1:**
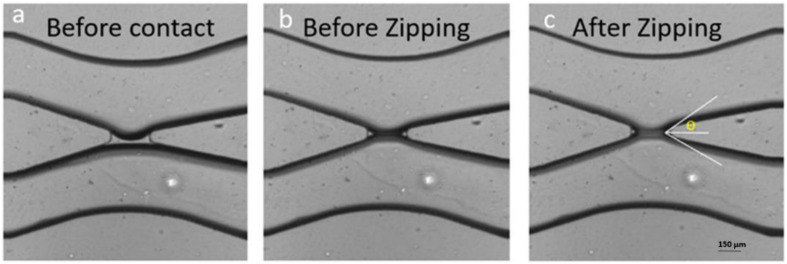
Formation of a bilayer lipid membrane (DOPC) in a microfluidic device. **(a)** The two interfaces of the buffer fingers in the top and bottom channel are decorated by lipid monolayers but are still separated by a thin oil layer present in the gap. **(b)** The two monolayers were brought in contact by microfluidic manipulation and touch each other but did not yet form a lipid bilayer. **(c)** After formation a bilayer the conductivity can be measured by electrophysiological measurements and the contact angle θ can be obtained optically.

Measurements of the surface tension γ were done applying the pendant drop technique using a commercial device (OCA 20, data physics). A droplet of the respective electrolyte solution was dispensed from a needle immersed in a cuvette that is filled with the studied lipid/oil composition containing also gA at a concentration of 1 nM. Different concentrations of silicone (0%, 0.1%, 0.3%, 5%, and 10%) were used to extract its effect on the surface tension (Table 1). The corresponding membrane tension Γ for each buffer/oil/lipid composition (5 mg/ml lipid concentration), can be calculated from these surface tension (Table 1) using the following equation Γ = 2γ cos θ, where θ is the contact angle extracted from optical micrographs as indicated in Figure 1 (Bibette et al., 1999).

**TABLE 1:** Measurements of the water/(Squalene-SiAR20) interface interfacial tension in presence of 5 mg/ml DOPC as function of silicone oil concentration in the surrounding squalene oil.

**Si AR20 (vol%)**	**0**	**0.1**	**0.30**	**5**	**10**
γ (mN/m)	80.2	0.880.07	0.720.07	0.700.07	0.710.07
θ (°)	401	28.51	25.51	25.51	25.51
Γ (mN/m)	12.20.1	1.550.1	1.290.1	1.260.1	1.280.1

### Electrophysiological Measurements

All measurements were done using a Patch Clamp amplifier (EPC 10 from Heka-Electronics). Two homemade Ag/AgCl electrodes were prepared by chlorination of ultra-clean Ag wires with diameter of 150 μm. The chlorination was achieved by immersing the two Ag wires in sodium hypochlorite solution (Merck, Germany) for about 30 min while applying a potential difference of a few volts. After chlorination; Ag wires turned into reference Ag/AgCl electrodes and we connected both of them to the probe of the patch amplifier. The two electrodes were inserted into the microfluidic chip via the two inlets of the aqueous phase, a few hundred microns away from the lipid bilayer. As excitation signal, we used a sinusoidal wave at a frequency of 10 kHz and an amplitude of up to 50 mV.

#### Formation of a Lipid Bilayer With Vertical Orientation

Before introducing the aqueous solution inside the chip, first the lipid/oil mixture (5 mg/ml lipid concentration) was injected into the chip and let there at rest for about 1 h to ensure complete hydrophobicity of the microfluidic channels. Then the aqueous solutions were injected in the chip through the inlets using a homemade hydrostatic pressure control consisting of two plastic syringes filled with the aqueous solution hanging on a laboratory stand while both can be moved up and down in small steps. The two syringes are open at their top, which enables adding ions, proteins or any other cargos. During the slow advancement of the two electrolyte fingers inside the microfluidic chip, both water/oil surfaces are decorated with lipid molecules; forming eventually two lipid monolayers. Once the two aqueous fingers appear close to the gap of the x-junction, we lower the height of both plastic syringes to slow down their advancement. When both aqueous fingers reached the gap region, they touch each other and shortly after form a lipid bilayer membrane (Figure 1; Vargas et al., 2014; Khangholi et al., 2020).

#### Formation of a Lipid Bilayer With Horizontal Orientation

All the lipid-oil mixtures are prepared with a concentration of 5 mg/mL in squalene oil. The lipids are dissolved in squalene via magnetic steering and let at 45°C for 3–4 h until completely dissolved. The free-standing bilayer formation method is based on a variant of the DIB method (Bayley et al., 2008). Two buffer fingers are dispersed in an oil-lipid phase. As the lipid molecules are amphiphilic, they are similar to a surfactant and they are covering each water-oil interface. Our chip is designed to position the two buffer fingers close to each other and only separated by an oil-lipid sandwich. The thickness of this oil-lipid phase is approximately a few tenths of micrometers. As the PDMS chip is porous it drains the oil phase separating the two buffer phases, which eventually brings the two lipid monolayers in contact to from a lipid bilayer. Experimentally, we perform this by filling the bottom microchannel by buffer first. The complete adsorption of squalene by PDMS walls varies between 1 and 2 h depending on the volume of lipid-oil mixture introduced to the system. After absorption of the oil, the lipid-oil sandwich becomes thinner and the lipid bilayer formation visually starts to be visible by the observation of a circle at the middle of the aperture between the two microfluidic channels.

### Single Channel Analysis

For Single channel analysis (Figure 6), Gramicidin A (Sigma-Aldrich) was added into both aqueous phases surrounding a bilayer. The concentration of gA was 1 nM and the buffer solutions contained 1 M NaCl to enable electrophysiological measurements of the expected ion channels. After letting the system equilibrate for 15–20 min to form a stable bilayer, the bilayer was caught with a micropipette. To investigate the conductive properties of possibly formed pores, a silver chloride electrode connected to a patch-clamp setup was inserted into the micropipette (EPC10 – HEKA Germany). Using the single channel analysis mode, we recorded the current through the voltage-clamped membrane. Only after dispersing gA into the aqueous phases, an unitary amplitude of ≈2.8 pA is recorded, see Figure 6 (solid black line). This value is characteristic for gA pores under the used experimental conditions. As a control test, this experiment was repeated, yet adding 5 × 10^–3^ M of calcium chloride (CaCl_2_) into the buffer phases. The presence of divalent ions, such as calcium, is known to block the conductive properties of gA, when inserted in planar lipid bilayers. The presence of the divalent ion CaCl_2_ blocked the conductive property of gA, inserted into a bilayer (as expected).

## Results and Discussion

We will first discus the transport properties of Gramicidin A pores without silicone oil and subsequently their altered transport properties in presence of silicone oil inclusions.

### Transport Properties of an Oil-Free Lipid Bilayer With Gramicidin A Pores

**FIGURE 2:**
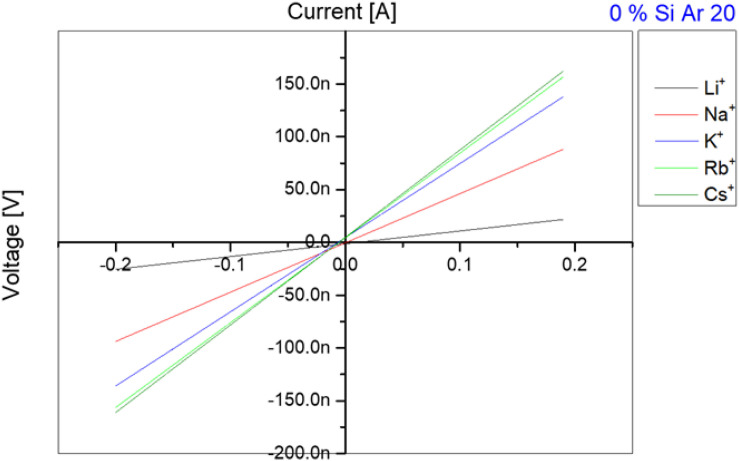
Current-voltage measurements for different ions crossing through gA ion channels inserted in a DOPC bilayer in absence of Si-oil. According to the atomic weight, the transfer velocity through gA ion channels is increasing with increase in atomic weight until reaching Rb^+^ and Cs^+^ which have a very close transfer rate and sometimes rates for Rb^+^ are faster than for Cs^+^.

From an electrical point of view, a pure lipid bilayer is equivalent to a dielectric, which makes it impermeable to charged molecules or ions (Miyamoto and Thompson, 1966). This is demonstrated by measuring the specific capacitance (∼ 3.7 mF.m^–2^ for DOPC; Vargas et al., 2014) of the bilayer formed in our microfluidic chip (Figure 1). Also, no significant current could be measured as function of an applied voltage, as long this voltage is below the well-known threshold voltage inducing electroporation. After dispersing gA monomers at both sides of the bilayer and waiting ∼10 min, electrophysiological measurements reveal a collapse of the capacitance signal and a current signal could be measured that is linearly dependent on the applied constant voltage (Figure 2). These characteristic signals demonstrate the successful reconstitution of gA ion pores in our oil-free free-standing lipid bilayer. Additionally, this type of pore is known to be blocked in presence of divalent calcium ions. Correspondingly, a few minutes after dispersing 20 mM CaCl_2_ buffer phase around the bilayer containing gA, we measured an increase of the capacitance signal close to the value obtained for a pure lipid bilayer and a simultaneous collapse of the current signal across the formed bilayer.

**FIGURE 3:**
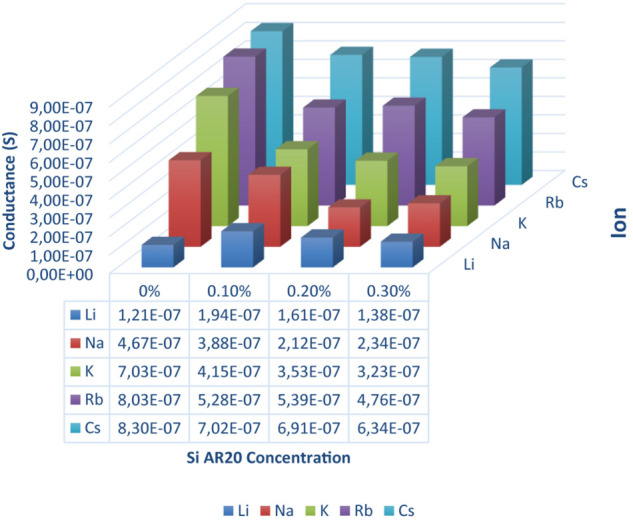
DOPC bilayer conductance measured in presence of gA ion pores for five different monovalent ions (Li, Na, K, Rb, and Cs) and four different silicone oil (Si AR20) concentrations up to 0.3 vol% in squalene oil. For this fairly low Si-oil concentration, we can notice that Li^+^ has the lowest conductance in all oil compositions, while Rb^+^ and Cs^+^ have the highest conductance. In general, there is a decrease in conductance for all ions after the addition of Si AR20 except for Li^+^ which has the lowest conductance. The most pronounced decrease in conductivity for increasing concentration of SiAR20 is observed for K^+^. Each point was on obtain on ∼100 measurements.

After this basic test that demonstrates the functionality of the gA pores inserted in our free-standing bilayer, we determine the transport properties of these pores for the following monovalent ions: Li^+^, Na^+^, K^+^, Rb^+^, Cs^+^ in a way that these measurements could be compared. For that we first produced a lipid bilayer as describe in section “Single Channel Analysis,” with a buffer that contains 1 nM of gA monomers and 100 mM of the respective salt. After 10 min of stabilization, we exchanged the buffer phase around the bilayer and replaced it with a buffer containing the desired ion types (for example Na^+^/Cl^–^) and without proteins. It is important to note, that this exchange of buffer is conducted using microfluidic pressure pumps whereas the lipid bilayer do not break during this procedure. Moreover, even the bilayer area stays about constant during this process and no noticeable change could be observed optically. After inserting the gA pores and adjusting the desired ion concentration, we measured a series of 10 characteristic current-voltage curves for each ion type and plotted the averaged results, as exemplary shown in Figure 2. The slope of each curve in Figure 2 is equal to the conductance of the bilayer, as plotted in Figure 3. Once such a measurement series is completed, we changed again the buffer phase around the bilayer containing another type of ionic charges. This protocol is repeated until we measured the current-voltage properties of the bilayer containing gA pores for the following five type of monovalent ions: Li^+^, Na^+^, K^+^, Rb^+^, Cs^+^. It results that for the same applied voltage we measured different fluxes for the different monovalent charges, respectively bilayer conductivities. In particular we obtained conductivities [Cs^+^] > [Rb^+^] > [K^+^] > [Na^+^] > [Li^+^] for the same studied Si-oil free lipid bilayer (Figure 3), which corresponds to the characteristic diffusion rate of these monovalent cationic charges through gA pores as reported in literature (Sandblom et al., 1977; Eisenman et al., 1978; Latorre and Miller, 1983).

### Transport Properties of Gramicidine A Pores Inserted in an Oil Containing Lipid Bilayer

To form a bilayer that contains silicone oil inclusions, we just mix a certain percentage of silicone oil to the squalene oil before forming a lipid bilayer. When the water-oil interfaces of the aqueous fingers coated with lipid molecules are brought into contact in such a surrounding oil phase, they form a bilayer containing some silicone oil as the dewetting process forming a bilayer is in general unable to expulse the silicone oil molecules completely. It results that this is a simple method to obtain a bilayer that contains some silicone oil molecules. The resulting trapped amount of oil is not easy to measure, but we can guess that the amount of trapped oil is proportional to the percentage of silicone oil that is originally contained in the squalene oil.

**FIGURE 4:**
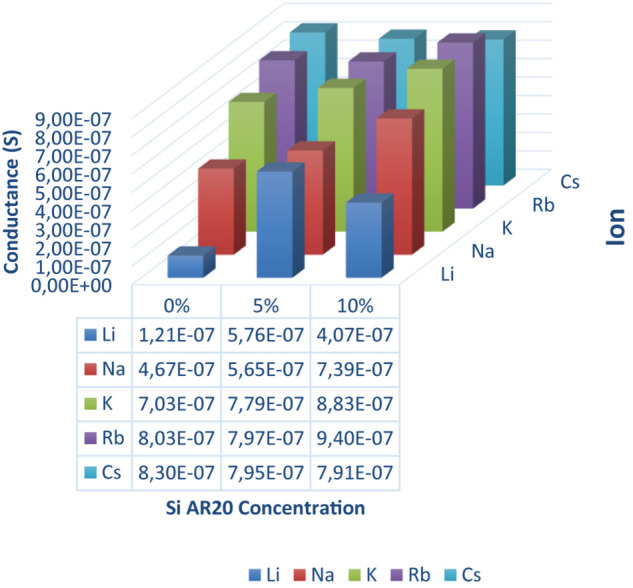
Conductance of a DOPC bilayer in presence of gA ion pores measured for five different monovalent ions (Li, Na, K, Rb, and Cs) and three different silicone oil (Si AR20) concentration up to 10 vol% in squalene oil. For this range of large Si-oil concentrations we can notice that Li^+^ has the lowest conductance in all oil compositions, while Rb^+^ and Cs^+^ have the largest conductance. In general, there is an increase in conductance for all ions after the addition of Si AR20 except in Li^+^ which has the lowest conductance. Each point was on obtain on ∼100 measurements.

**FIGURE 5:**
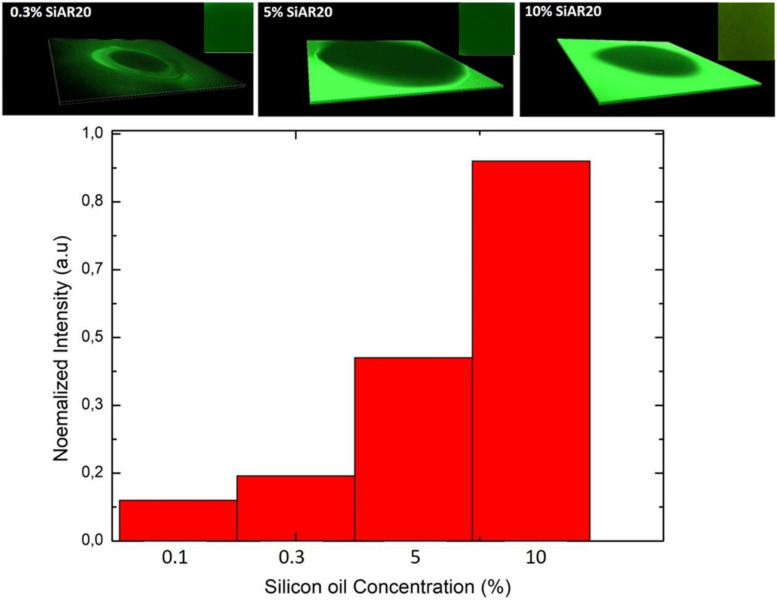
**Upper row:** 3D representation of a fluorescence inspection of a horizontal free-standing DOPC bilayer with a confocal microscope; inset shows a region of interest of the bilayer from the top view. The silicone oil is labeled with BODIPY and the bilayer picture is obtained after its zipping. The highly fluorescence region of the green area indicates the squalene/silicone oil reservoir. The green signal with a less intense fluorescence in the middle shows the bilayer region for a define concentration of silicone oil. **Lower row:** Normalized intensity signals averaged over the bilayer region of interest after normalized their intensity signal in respect to the strongest signal.

Adding (0.1–0.3%) silicone oil in a lipid bilayer affects the physical properties of this bilayer in several ways. The bilayer tension is affected by the presence of silicone oil. It is known that silicone oil stabilizes lipid bilayer by reducing the corresponding bilayer tension (Thiam et al., 2012; Guo et al., 2018). Measurements of the surface tension of a lipid coated water-(Squalene – silicone oil) oil interface and calculations of the bilayer tension based on the optically obtained bilayer contact angle θ (cf. Figure 1), confirm a drastic reduction of the bilayer tension (Table 1) and thus an increase of bilayer thickness (Goulian et al., 1998). However, it is commonly believed that silicone oil is weakly inserting into the lipid bilayer for this range of silicone oil concentration (Guo et al., 2018). In fact, a weak presence of silicone oil can be demonstrated via fluorescence inspection by confocal microscopy (Figure 4). Using an oil tracer (BODIPY), the presence of an oil is demonstrated by measuring a weak fluorescent signal in our lipid bilayer (Figure 5; Heo et al., 2019). Inspecting our bilayer, the shape of individual oil inclusions could not be characterized, which means that individual inclusions must be smaller than the lateral resolution of about 250 nm and can therefore not be resolved by confocal microscopy. This means that we have nano-inclusions and certainly no micro-inclusions. It is important to note that without the presence of silicone oil, no fluorescent signal is visible into the bilayer (Heo et al., 2019).

Adding (5–10%) silicone oil molecules in our lipid bilayer, we do not measure a change of surface tension compared to (0.1–0.3%) silicone oil (Table 1). This means that the membrane tension stays constant despite the increased silicone oil inclusions in our lipid bilayer. However, comparing specific capacitance measurements (∼ 3.7 mF.m^–2^ of a protein-free DOPC bilayer to the value of ∼ 3 mF.m^–2^ for 10 vol% silicone oil reveal an increase by about ∼1 nm), which corresponds to an increase about ∼20% of the bilayer thickness. The presence of silicone oil is also clearly visible via fluorescence inspection (Figure 5) for 5% and 10%vol. Interestingly, individual oil inclusions could not be resolved which means that individual inclusions must be smaller than the lateral resolution of about 250 nm and can therefore not be seen. These electrophysiological and optical results indicate that silicone oil inserts strongly into the bilayer core at these large Si-oil concentrations. Thus, we can safely assume that the bilayer thickness increases for (5–10%) silicone oil in comparison to small silicone oil concentrations of (0.1–0.3%).

Now, we can measure the transport properties of gA pores as function of the nature of the ionic charges (Li^+^, Na^+^, K^+^, Rb^+^, Cs^+^). To measure the different transport properties of gA for these ions, we follow the procedure described in section “Single Channel Analysis,” with a buffer that contains gA monomers. Then, after ∼10 min, we exchanged the buffer phase around the bilayer by a buffer containing one type of ionic charges (for example Na^+^/Cl^–^) and no proteins. Then we measured a series of 10 characteristic current-voltage curves and plot the average results, as shown in Figure 2. Once this step is conducted, we changed again the buffer phase around the bilayer with another type of ionic charges and repeat the same measurement for this type of ionic charge. These measurements were repeated for fairly small silicone oil concentrations of 0.1 vol%, 0.2 vol%, and 0.3 vol% and we measured a transport rate through gA pores that is following the relation [Cs^+^] > [Rb^+^] > [K^+^] > [Na^+^] > [Li^+^] like in a bilayer without silicone oil (Figure 3). Compared to the case without silicone oil, the ionic transport rate seems to be reduced when adding a little silicone oil. This point could be understood as a reduction of pore conductivity due to a decrease of membrane tension from ∼8 to ∼1 mN/m that is accompanied with an increase in bilayer thickness. Because the thickness of a gA dimer is less than the thickness of a relaxed bilayer (when the bilayer is not under tension), a gA dimer locally compresses the bilayer (Goulian et al., 1998). However, when the bilayer is under high tension, i.e., bilayer tension >4 mN/m, the bilayer thickness becomes close to the extension of a gA dimer. Therefore, it is easier to form gA pores and so the bilayer conductivity is increased (Goulian et al., 1998).

**FIGURE 6:**
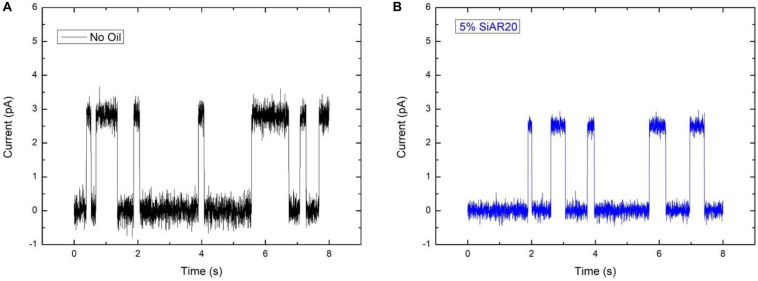
Ionic conductance of a single gA pore embedded in a DOPC bilayer, **(A)** without silicone oil and **(B)** with 5 vol% silicone oil AR20 in the squalene phase. The buffer contains 1 M NaCl, the applied potential difference across the bilayer is 200 mV, T = 25°C. The conduction of gA without Si-oil in **(A)** is around 2.8 pA and **(B)** around 2.5 pA in the presence of Si-oil. Measurements present, here, are a short measurements on hundreds of reproducible single jumps.

If the amount of silicone oil is raised to larger concentrations, of 5 vol% or 10 vol%, the measured transport rates are no more respecting the characteristic transport rate dependence for ions across gA pores. For those large silicone oil concentration of 5 vol% and above, we even obtained a conductance that is about equal for [Cs^+^] ∼ [Rb^+^] ∼ [K^+^], which is characteristic for a saturation of the channel via, maybe, a single-file transport of these cations (Finkelstein and Andersen, 1981). We can even suppose that the reduction of membrane tension due to inserted silicone oil, is reducing the pore size and facilitates the saturation of the gA ion pore via a possible single-file transport (Finkelstein and Andersen, 1981; Goulian et al., 1998). To test this hypothesis, we performed single-channel analysis for a DOPC bilayer containing gA ion pores without silicone oil, and with 5 vol% silicone oil in the surrounding squalene phase (following the method describe by Hähl et al., 2017) (Figure 6). It results that these measurements reveal a slight reduction of a single-channel conduction from ∼2.8 pA for a bilayer without oil inclusions to ∼2.5 pA in presence of silicone oil (Figure 6), with $r^{2}=\frac{l.\Delta \,I}{\pi \,G\,C\,U}$   (where *r* is the pore diameter, *l* is the length of the channel, Δ*I* is the step increase in current, *G* is the molar conductivity, *C* is the concentration, and *U* is the applied voltage). This fact could be understood as a reduction of the pore radius due to an increase of the bilayer thickness which is facilitating the possibility of single-file cation transport (Lundbaek and Andersen, 1994; Goulian et al., 1998; Lundbaek et al., 2010).

## Conclusion

We studied the transport properties of Gramicidin A in a heterogeneous lipid bilayer that was fabricated using a microfluidic scheme. We studied how silicone inclusions are affecting the transport properties of gA. It results that without silicone oil, the gA pores exhibit the transport properties reported in the literature. However, with increasing silicone oil inclusion (0.1–0.3) vol% in the bilayer, the transport properties of gA pores embedded in these bilayers is reduced with respect to their standard behavior. For the largest silicone oil concentration tested (5 vol% and above), the conductivity seems to saturate on a increase level and we even obtain about equal transport properties for [Cs^+^] ∼ [Rb^+^] ∼ [K^+^], which is characteristic of a saturation of the gA channel via, maybe, a single-file transport of these cations. We suppose that this effect is due the reduction of membrane tension that is induced by the presence of silicone oil that is increasing the bilayer thickness, which is reducing the pore size and thus facilitate the channel saturation. This hypothetic channel pore reduction is supported by single-channel analysis performed in presence of 5 vol% silicone oil. Thus, even that we demonstrated that silicone oil can affect the transport properties of ion channels. The oil quantities needed to perturb the ion channel transport remarkably, are too high to be considered as biologically relevant.

## Data Availability Statement

The datasets generated for this study are available on request to the corresponding author.

## Author Contributions

HT, SP, and J-BF performed the measurements. RS and J-BF directed the research. J-BF designed the research. All authors contributed to the article and approved the submitted version.

## Conflict of Interest

The authors declare that the research was conducted in the absence of any commercial or financial relationships that could be construed as a potential conflict of interest.
