# Supplementary material for: Transport Properties of Gramicidin A Ion Channel in a Free-Standing Lipid Bilayer Filled With Oil Inclusions
Source: Front Cell Dev Biol. 2020 Sep 4;8:531229. doi: 10.3389/fcell.2020.531229 (PMC7498540; doi:10.3389/fcell.2020.531229)
Supplement: Supplementary file 1 [file Image_1.pdf]

Supplementary Information: Transport properties of Gramicidin A ion channel in a free-standing lipid bilayer filled with oil inclusions

Harvey Tawfik, Sevde Puza, Ralf Seemann, Jean-Baptiste Fleury*

Universitat des Saarlandes, Experimental Physics and Center for Biophysics, 66123 Saarbruecken, Germany

*** Correspondence:**
jean-baptiste.fleury@physik.uni-saarland.de

2D - Microfluidic Chip Fabrication (for vertical bilayer production):

We are producing the 2D microfluidic Chip by using photolithography. Firstly, the 2-inch silicon wafer is cleaned by acetone and ethanol, and rinsed with deionized water. Then baked on a hot plate at 95°C for 5 minutes to have a clean and uniform substrate for photoresist coating. The spinning speed and the time of the spin coater is determined to end up with a final coating thickness of 100 µm on the substrate. 5 to 10 mg of SU-8 is put on the silicon wafer and placed in the Spin-Coater. Acceleration is set to 500 rpm for 20 seconds, then 1500 rpm at 300 rpm/second for 60 seconds to cover the entire surface uniformly with photoresist. Prior to UV exposure, the photoresist is pre-baked for 1 minute at 65°C and then soft baked for 5 minutes at 95◦C. To prepare the photoresist coated silicon wafer for UV exposure, a transparent mask with the pattern of the desired bottom channel was placed on it. Afterwards, exposed to UV light with a wavelength of 400 nm with the intensity of 15 mW/cm^2 for 20 seconds. Following that, post exposure step is done by baking the photoresist at 65◦C for 1 minute and then 95°C for 5 minutes to obtain the selective cross-linking. Then, the substrate is immersed in developer solution and gently shaken for 10 minutes to dissolve the non-exposed areas properly. As the last step, the substrate is rinsed with acetone, ethanol and distilled water before using it as a master for the microfluidic device production. PDMS (Sylgard 184 - DowCorning) is poured on the structured silicon wafer with a ratio 10:1 (polymer:cross linker). The PDMS is degassed for 30 minutes and then and cured at 100°C in a oven. Then the cross linked PDMS chip is removed from the silicon wafer. Holes are punched into the PDMS chip to form inlets and outlets before being covalently bond with a glass coverslip via a plasma treatment (Diener electronics). An homemade syringe-micropump is, then, connected to the chip using PTFE (Teflon) tubing (Fig. S1-S2).

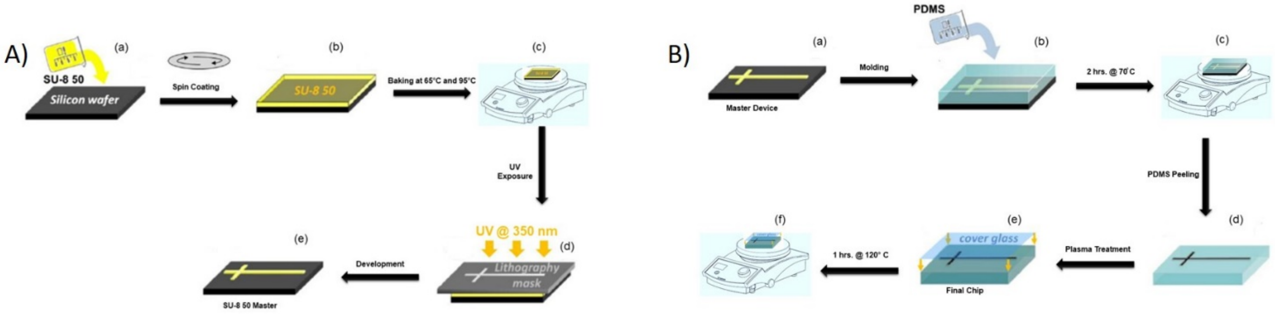

Figure S1: A) Soft photolithography procedure (a) addition of SU-8 50 to a pre-cleaned silicon wafer (b) after spin coating, the SU-8 is uniformly distributed (c) soft baking to evaporate the solvent from the photoresist (d) the mask is on top of the silicon wafer then starting UV exposure (e) the final SU-8 master after development. B) PDMS fabrication (a) Cleaned SU-8 50 master prepared by photolithography (b) addition of PDMS mixture to the master (c) baking of the PDMS mixture (d) the PDMS is peeled from the master (e) plasma treatment for PDMS and glass followed by attaching both part to each other (f) heating the chip to insure irreversible bonding.

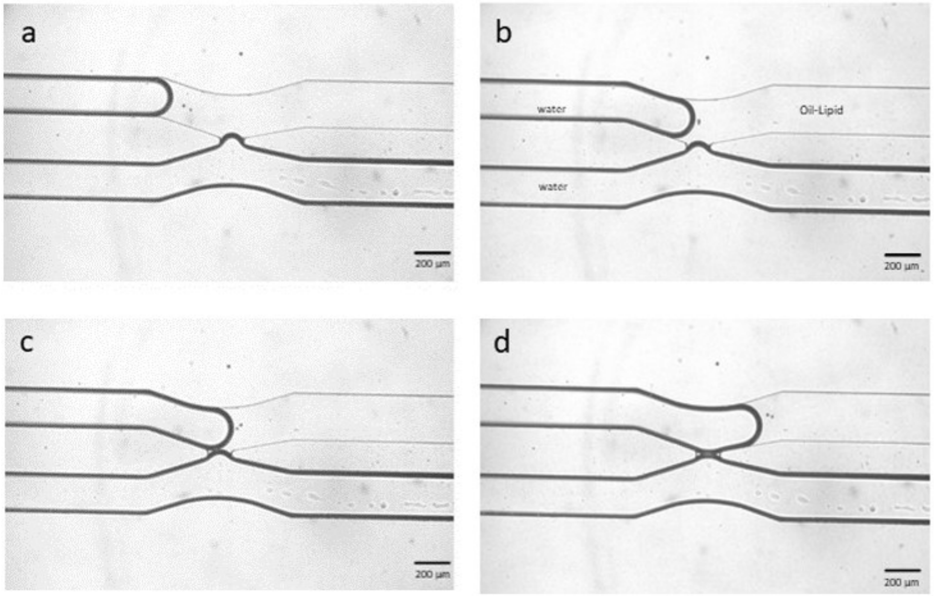

Figure S2: Design of the 2D microfluidic chip and formation of a lipid bilayer at a define position (from a to d).

3D-Microfluidic Chip Fabrication (for horizontal bilayer production):

For produce the 3D Chip, we use a 3D-Printing technique to have a 3d mask for the production of a PDMS microfluidic chip. The technique is producing a mold using a 3D printer ultimaker 3 (Ultimaker), and PLA (polylactic acid) as a polymer filament (2.85 mm diameter) purchased from Ultimaker. Using a nozzle of 250 µm (from Ultimaker), we deposit the polymer via the thermoprinting technique (at 230°C) on a glass surface, following a 3D-design drawn using the software Autocad. A PLA mold is produced in less than 1 hour, and a picture of this mold is presented at the top panel of Fig. S2. Then PDMS (Sylgard 184 – DowCorning) is degassed for 30 minutes and poured directly into the system and cured at 55°C for 2 hours in an oven. After the PDMS is fully cured, the cone of the 3D-printed structure is broken and carefully removed from the PDMS microchip. This step creates the upper conic hole in the microfluidic device. Afterwards, the cured PDMS is detached from the PLA mold, and the device has obtained its final shape of one channel connected to a conic hole that is open to the air. Inlet and outlet holes are punched, and the PDMS microchip is then bonded to a thin glass coverslip using plasma bonding (Diener electronics). The final device is put on a hot plate at 95°C for 1 hour to increase the bonding strength. A homemade syringe-micropump is, then, connected to the chip using PTFE (Teflon) tubing (Fig. S3).

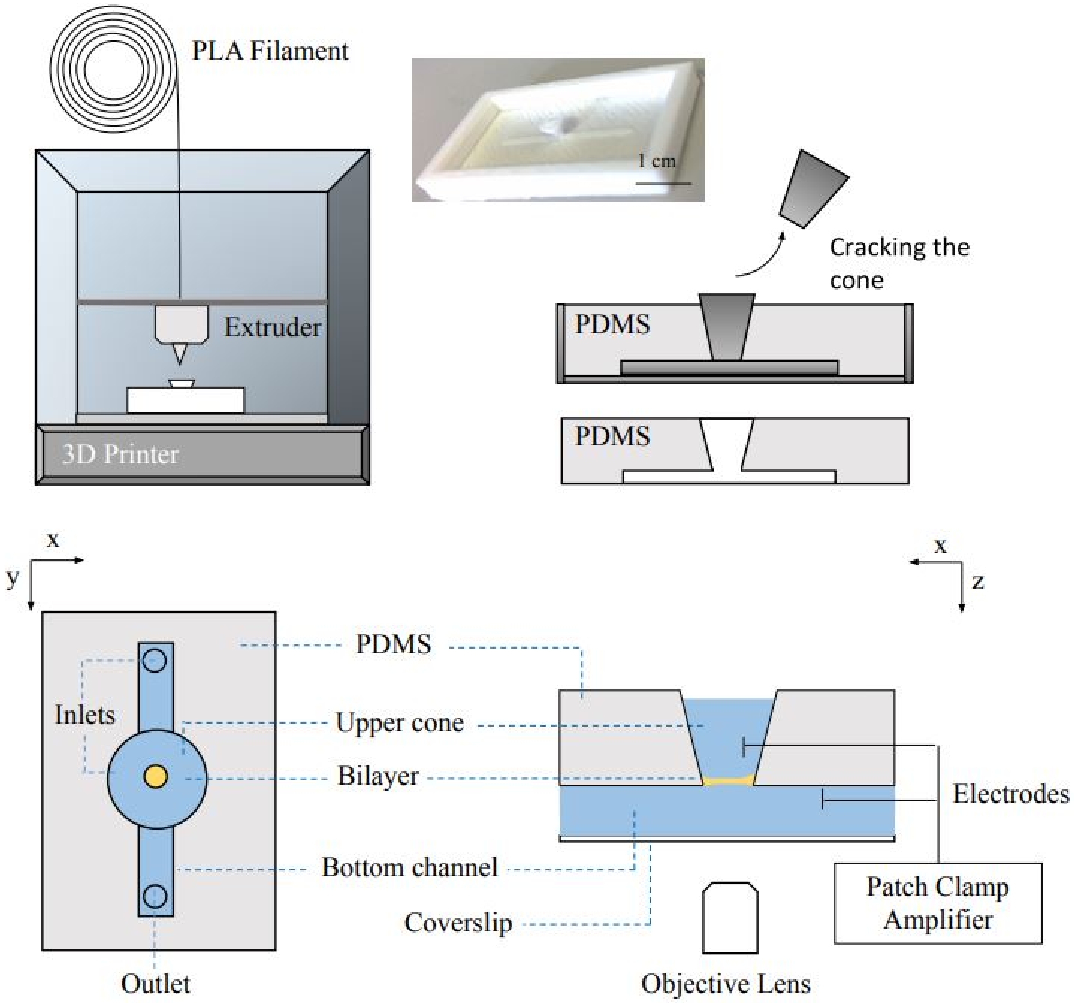

Figure S3: Scheme presenting the microfluidic chip production from the mold production using a 3D-printer and the PDMS microfluidic chip design after.
